# Researphobia and the production of evidence: a reflexive perspective on researching sexual and gender minority populations in South Africa

**DOI:** 10.3389/fpubh.2026.1988663

**Published:** 2026-10-21

**Authors:** Lebogang Manthibe Ramalepe

**Affiliations:** COMPRES Research Entity, School of Psychosocial Health, Faculty of Health Sciences, North-West University, Vanderbijlpark, South Africa

**Keywords:** epistemic injustice, global south, hard-to-reach populations, LGBTQI+, reflexivity, research ethics, sexual and gender minority health, South Africa

## Abstract

Sexual and gender minority (SGM) populations in the Global South are routinely described as hard to reach, yet the conditions that make them difficult to recruit are the same structural conditions that generate their health disadvantage. This Perspective examines the production of evidence rather than its content, drawing on a doctoral mixed-methods study of substance use, perceived discrimination, and social support among SGM individuals in South Africa. I argue that recruitment friction is data. Gatekeeping organisations that declined to circulate the survey without participant compensation were advancing a defensible claim to reciprocity on behalf of over-researched communities; widespread non-response reflected research fatigue and warranted scepticism toward time-limited academic engagement rather than simple unavailability. Digital snowball recruitment substantially outperformed institutional routes, but carried a bias that is rarely stated in life-course terms: it privileges younger, urban, digitally connected, and already-disclosed participants, and systematically under-recruits the older, rural, and concealed populations about whom the least is known. I further introduce researphobia to name the institutional and epistemic hostility directed at scholarship centring lesbian, gay, bisexual, transgender, queer/questioning, and intersex (LGBTQI+) lives, expressed through funding and publication barriers and through everyday interactions that delegitimise both the work and the researcher. Read through the lens of epistemic injustice, researphobia raises the cost of entry into a field where African-generated evidence is already scarce, and so contributes directly to the gaps it is then used to justify. Alongside these constraints, the study demonstrated the value of relational ethics, sustained reflexivity, and community-rooted enquiry. I close with implications for researchers, ethics committees, and funders working with sexually and gender diverse populations in the Global South.

## 1 Introduction

Research with hard-to-reach populations sits at the intersection of methodological difficulty and ethical obligation (1, 2). “Hard-to-reach” is itself a contested designation: populations are rarely inherently inaccessible, but are rendered so by criminalisation, stigma, institutional surveillance, and the accumulated memory of extractive research (3). For sexual and gender minority (SGM) communities in the Global South, this designation carries a further irony. The very structures that make these populations difficult to reach are the structures that produce their health disadvantage, so the methodological problem and the substantive problem are the same problem.

The evidence base unambiguously shows that SGM populations carry a disproportionate burden of substance use and mental health morbidity, and that this burden is patterned by stigma, minority stress, and structural discrimination rather than by identity itself (4–6). What is far less developed is an account of how that same stigma structures the production of evidence: who gets counted, who is willing to be counted, and which researchers are permitted to do the counting. Where such accounts exist, they are frequently written from the Global North and imported wholesale, reproducing the Eurocentric framing that African scholarship has long contested (7–9). Reimagining SGM research in African settings requires more than translating instruments; it requires interrogating the conditions under which knowledge about these communities can be generated at all (10).

This Perspective offers such an account. It draws on my doctoral study of the role of substance use in the relationship between perceived discrimination and social support among SGM individuals in South Africa, which used a sequential explanatory mixed-methods design: a quantitative survey followed by qualitative interviews that contextualised and complicated the survey findings (11). Rather than reporting those findings, I examine the conditions of their production. I argue three things. First, that gatekeeping, compensation disputes, and non-response are not incidental frictions but data about the relationship between marginalised communities and the research enterprise. Second, that the recruitment strategies that succeed carry their own systematic biases, with consequences for what the evidence base can say about health inequities across the life course. Third, that resistance to this scholarship operates inside the academy as well as outside it, a phenomenon I name researphobia. I close with transferable implications for researchers, ethics committees, and funders working with sexually and gender diverse populations in the Global South.

## 2 Positionality and context

Reflexive scholarship requires that I locate myself. I write as a Black South African woman, a psychologist, an LGBTQI+ researcher, and an advocate. These positions are not separable, and their friction is generative rather than incidental. Advocacy is sustained by proximity and moral urgency; research demands distance, disconfirmability, and a willingness to publish findings that complicate the advocacy position. Holding both means entering institutional and social spaces that are hostile to LGBTQI+ people in order to produce the evidence that inclusive policy requires.

The South African context is one of sharp contradiction. Constitutional protection on the grounds of sexual orientation coexists with entrenched homophobia and transphobia in families, healthcare facilities, workplaces, schools, and universities (12–14). Colonial-era claims that same-sex desire was foreign to Africa continue to shape contemporary discourse, and the resulting climate means that legal protection and lived safety diverge substantially (13). This divergence is visible in health systems: heteronormative assumptions are embedded in intake procedures, clinical training, and provider attitudes across African healthcare settings, producing avoidance, delayed presentation, and identity concealment among LGBTQI+ patients (15, 16). A community that has learned to conceal from clinicians has good reason to conceal from researchers. Recruitment difficulty is therefore not a methodological accident; it is the predictable downstream effect of institutional heteronormativity, and it is compounded for participants whose marginalisation is multiply constituted by race, class, gender identity, and geography (17).

## 3 Researphobia: epistemic resistance inside the academy

Alongside the hostility that participants encounter, there is a form of resistance directed at the research itself. I use the term *researphobia* to describe institutional and epistemic hostility toward scholarship that centres LGBTQI+ lives, expressed through the devaluation of the work and the delegitimation of the researcher who conducts it.

Researphobia operates at two levels. Structurally, it appears as difficulty in securing funding, scepticism from reviewers unfamiliar with the field, the framing of SGM health as niche rather than population health, and the absence of the topic from curricula. Interpersonally, it appears in remarks that seem casual and are not: being asked why one is “interested in gay people’s research,” being told that LGBTQI+ concerns are exaggerated, or being met, on introducing the study, with the question “Are you gay?” That last question does specific work. It converts a scholarly contribution into a personal disclosure and implies that only an insider would pursue the topic, which simultaneously outs the researcher and discredits the research.

This is usefully read as epistemic injustice: testimonial injustice, where a knower is accorded diminished credibility because of prejudice about their identity, and hermeneutical injustice, where a community lacks the shared conceptual resources to make its experience intelligible (18). Naming researphobia is an attempt to supply one such resource. It is also consistent with what is known about African workplaces more broadly, where heteronormativity is reproduced through informal culture and everyday interaction rather than through formal policy alone (19). The academy is a workplace, and it is not exempt. The consequence is not merely discomfort for individual scholars; it is a distorted evidence base, because researphobia raises the cost of entry into a field where African-generated knowledge is already scarce (20).

## 4 Researchers Reflective notes

### 4.1 Reflective note one: gatekeeping, reciprocity, and non-response

Recruitment proceeded through two channels: LGBTQI+ civil society organisations (CSOs), which were approached by email, and social media distribution with snowball referral. The CSO route is conventionally recommended because organisations hold community trust that researchers have not earned (21, 22). It largely failed in two instructive ways.

First, several organisations asked whether participants would be compensated. When I explained that no incentive was available because of budgetary constraints, a number declined to circulate the survey. It is tempting to read this as an obstacle. It is more accurately read as a legitimate position advanced by organisations serving communities that are over-researched and under-compensated, and that have watched research careers advance on the basis of their members’ disclosures without discernible return (23). The literature on participant payment is genuinely unsettled, weighing improved participation and recognition of participants’ time against concerns about undue inducement and distributive fairness (24). My study did not resolve that tension; it simply lacked the budget to enter the debate, which is itself a structural finding. Under-resourced researchers in the Global South are disproportionately unable to offer reciprocity, and their studies are therefore disproportionately excluded from precisely the communities most in need of evidence.

Second, most CSOs did not respond at all. Non-response is easily coded as absence and easily dismissed. It is better read as a signal: overstretched organisations, research fatigue, and reasonable scepticism toward academics who arrive at the point of data collection and depart at the point of publication (7, 23). The corrective is not a better email. It is a different temporal relationship with communities, closer to the sustained partnership models of community-based participatory research, in which engagement precedes the study and outlives it (25, 26). Ethics committees and funders have a role here: consent procedures are scrutinised closely, while reciprocity, dissemination back to communities, and the sustainability of partnership are seldom assessed at all.

### 4.2 Reflective note two: digital recruitment and its life-course blind spots

Social media recruitment, using a digital poster and snowball referral through Facebook, X, WhatsApp, and Instagram, substantially outperformed the CSO route. Online snowballing is well established for accessing hidden populations, and its advantages here were anonymity, participant control over the pace and terms of engagement, and the peer endorsement carried when material arrives from a known contact rather than an institution (21, 27, 28). Digital platforms have become significant sites of connection, information-seeking and health-related engagement, particularly where offline visibility carries risk (29, 30). For a study asking about discrimination, substance use, and identity, that combination of reach and distance mattered.

The success came at a cost that reflexive practice obliges me to state plainly. Snowball referral through digital networks systematically favours those who are digitally connected, familiar with the platforms, and already embedded in a network in which the topic can be discussed. It skews younger, more urban, better resourced, and more disclosed. It under-recruits older SGM adults, rural participants, those without stable data access, and those whose concealment extends to their online presence. Because minority stress accumulates across the life course, and because coping repertoires and support structures differ markedly between a 22-year-old in Johannesburg and a 55-year-old in a rural district, this is not a peripheral limitation. It means that the sampling frames most accessible to under-resourced researchers are biased against exactly the life stages and geographies where evidence on SGM health inequities is thinnest. Convenience in recruitment translates directly into a truncated life-course picture, and the field should treat this as a substantive threat to inference rather than a routine disclaimer in a limitations paragraph.

### 4.3 Reflective note three: trust, rapport, and relational ethics

Trust could not be assumed. Many SGM South Africans have encountered clinical, psychological, and academic settings as sites of surveillance, misrecognition, or harm (14, 16, 31). Approaching such communities as a researcher, however well-intentioned, reasonably invites scepticism. I was not asking participants for data; I was asking them to be vulnerable in a context that has repeatedly told them their experience is invalid.

Practically, this meant affirming language in all materials, self-identification options beyond binary categories, explicit and repeated confidentiality assurances, and transparency about my own positionality and commitments. Disclosing that investment carries professional risk, but it disrupted the subject–investigator hierarchy and moved participants closer to collaborators in knowledge production (32). In the qualitative phase, where participants were asked to revisit discrimination, substance use, and support, I routinely began by talking and listening without reference to the schedule and allowed participants to set the pace. Several described the interview as a rare space to speak without judgement, which is a reminder that a well-conducted interview can hold space as well as extract information (33).

Trust-building also failed at times, and those failures were instructive. Hesitation about how narratives would be used, and whether anonymity would truly hold, surfaced repeatedly. These were ethically important moments in Guillemin and Gillam’s (34) sense: junctures where formal approval offered no guidance and judgement had to be exercised in real time. Sustained reflexivity about my own assumptions, power, and presence was the only available instrument (35). This work is also emotionally costly. Researching stigma while belonging to the stigmatised group is emotion work, and it is unevenly distributed among early-career and insider researchers who are least protected against it (36, 37). Institutions that expect this labour without resourcing debriefing, supervision, or psychological support are externalising a cost to the scholars least able to absorb it.

## 5 Opportunities and insights

The account above should not be read as damage-centred (8). The study generated substantial opportunity alongside difficulty. Engaging directly with participants surfaced the density of care, resistance, and solidarity that characterises queer life in South Africa, and the role of informal support networks in buffering the effects of structural discrimination on substance use and mental health, a complexity that is routinely flattened in global discourse about African sexualities (38). Methodologically, the sequential design demonstrated its value precisely where qualitative narratives contradicted rather than confirmed the survey pattern, which is the condition under which mixed methods earn their cost. The process also clarified reflexivity as a daily practice of accountability rather than a paragraph in a methods section (39). Finally, the study reinforced that advocacy-oriented research and methodological rigour are not in tension, and that this integration is increasingly recognised in regional and multi-country health research agendas addressing gender-based violence, sexual and reproductive health, and the health of marginalised populations (40).

## 6 Discussion

Three implications follow for research practice with sexually and gender diverse populations in the Global South. Table 1 summarises the difficulties encountered, how I now read them, and the practices they imply. For researchers, recruitment difficulty should be documented and analysed rather than compressed into a sentence about response rates. Who declined, who did not answer, and what was asked of the researcher in exchange for access constitute findings about the community–research relationship. Sampling limitations should be stated in life-course and geographic terms, not only in terms of sample size. For ethics committees, review protocols remain oriented toward institutional risk and individual consent, and are largely silent on reciprocity, dissemination, and the duration of engagement. Committees reviewing research with over-researched communities should require an account of what the community receives, and should recognise that researcher wellbeing is an ethical matter and not a personal one (41). For funders and institutions, the compensation gap is a structural equity issue. When budgets in Global South institutions cannot support participant reimbursement, community partnership, or translation and dissemination, the resulting evidence gaps are attributed to the difficulty of the population rather than to the underfunding of the research. Dedicated support for SGM health research in African settings, and for the scholars who conduct it, is the most direct available remedy for researphobia. This Perspective has clear limitations. It reflects the experience of a single researcher in one country, working in one methodological tradition, and it makes no claim to representativeness. Reflexive accounts are also vulnerable to retrospective coherence, in which uncertainty at the time is narrated as insight afterwards. The concept of researphobia is offered as a starting point that requires empirical development, including systematic study of funding, publication, and citation patterns for SGM-focused research in African institutions. I would welcome its contestation as much as its adoption.

**Table 1:** Difficulties encountered in researching sexual and gender minority populations in South Africa, their reinterpretation, and the practices they imply.

Encountered difficulty	How I read it in hindsight	Transferable practice
Civil society gatekeepers requested participant compensation.	A legitimate claim to reciprocity from over-researched, under-compensated communities, not an obstacle.	Budget for reciprocity at the proposal stage; where funds are unavailable, negotiate non-financial return (training, co-authorship, community reports) before approaching gatekeepers.
The majority of organisations did not respond.	Research fatigue, capacity constraints, and reasonable scepticism toward time-limited academic engagement.	Build relationships before there is a study to recruit for; report non-response as a finding rather than as attrition.
Social media snowballing outperformed institutional routes.	Peer endorsement and participant-controlled anonymity outweighed institutional credibility.	Use digital recruitment deliberately, but pair it with offline routes to reach non-connected participants.
The sample skewed young, urban, digitally connected, and disclosed.	A structural bias with life-course consequences, not a routine limitation.	State sampling limits in life-course and geographic terms; treat the gap as a research priority, not a caveat.
Participant hesitation about anonymity and data use.	Ethically important moments requiring in-the-moment judgement beyond formal approval.	Treat consent as ongoing; document micro-ethical decisions as part of the audit trail.
Dismissive and intrusive questioning from academic colleagues.	Researphobia: testimonial and hermeneutical injustice operating within the institution.	Name it; build peer and mentorship structures; resource debriefing and supervision for researchers studying their own communities.

## 7 Conclusion

Research with SGM communities in South Africa is methodologically demanding, ethically complex, and emotionally costly, and the difficulties are informative rather than merely inconvenient. Gatekeeping, compensation disputes, non-response, and the biases of digital recruitment are the fingerprints of the same structural inequities that produce SGM health disadvantage in the first place, and they shape which parts of the life course the evidence base can see. Researphobia names an additional barrier that operates inside the academy, raising the cost of doing this work where it is most needed. Making this research possible will require reciprocity built into the design rather than added at the end, ethics review that asks what communities receive, funding that reaches Global South institutions, and academic environments in which studying LGBTQI+ lives is treated as ordinary population health scholarship. Until then, reflexivity remains both a methodological obligation and a form of resistance.

## Data Availability

The original contributions presented in the study are included in the article/supplementary material, further inquiries can be directed to the corresponding author.
